# Minimum Leaf Conductance (*g*_min_) Is Higher in the Treeline of *Pinus uncinata* Ram. in the Pyrenees: Michaelis’ Hypothesis Revisited

**DOI:** 10.3389/fpls.2021.786933

**Published:** 2022-01-24

**Authors:** Amauri Bueno, David Alonso-Forn, José Javier Peguero-Pina, Aline Xavier de Souza, Juan Pedro Ferrio, Domingo Sancho-Knapik, Eustaquio Gil-Pelegrín

**Affiliations:** ^1^Chair of Botany II – Ecophysiology and Vegetation Ecology, Julius von Sachs Institute of Biological Sciences, University of Würzburg, Würzburg, Germany; ^2^Unidad de Recursos Forestales, Centro de Investigación y Tecnología Agroalimentaria de Aragón, Zaragoza, Spain; ^3^Instituto Agroalimentario de Aragón -IA2, CITA-Universidad de Zaragoza, Zaragoza, Spain; ^4^Aragon Agency for Research and Development (ARAID), Zaragoza, Spain

**Keywords:** cuticular waxes, minimum leaf conductance, Michaelis’ hypothesis, treeline, *Pinus uncinata*

## Abstract

The search for a universal explanation of the altitudinal limit determined by the alpine treeline has given rise to different hypotheses. In this study, we revisited Michaelis’ hypothesis which proposed that an inadequate “ripening” of the cuticle caused a greater transpiration rate during winter in the treeline. However, few studies with different explanations have investigated the role of passive mechanisms of needles for protecting against water loss during winter in conifers at the treeline. To shed light on this, the cuticular transpiration barrier was studied in the transition from subalpine *Pinus uncinata* forests to alpine tundra at the upper limit of the species in the Pyrenees. This upper limit of *P. uncinata* was selected here as an example of the ecotones formed by conifers in the temperate mountains of the northern hemisphere. Our study showed that minimum leaf conductance in needles from upper limit specimens was higher than those measured in specimens living in the lower levels of the sub-alpine forest and also displayed lower cuticle thickness values, which should reinforce the seminal hypothesis by Michaelis. Our study showed clear evidence that supports the inadequate development of needle cuticles as one of the factors that lead to increased transpirational water losses during winter and, consequently, a higher risk of suffering frost drought.

## Introduction

The alpine treeline determines the forest altitudinal limit. The transition from the sub-alpine forest to the alpine tundra, also known as the “tension zone” (Griggs, 1934), is formed by the forest mass opening, leading to isolated specimens. These specimens develop forms away from the typical tall, erect timber-sized tree as they approach the altitudinal limit of distribution (Wardle, 1965, 1968). Exposed trees frequently have the characteristic “flagged” crown structure shaped by continual exposure to freezing winds, which becomes progressively more stunted, culminating in prostrate individuals, called Krummholz (Marr, 1977).

The search for a universal explanation of this altitudinal limit for tree occurrence has given rise to different hypotheses, including specific physiological causes associated with the harsh conditions suffered by shoots (see Fernández et al., 2017 and references therein). During winter, the water uptake is limited by low soil temperatures, while the warm atmosphere and solar radiation increase evaporative demand, producing what is known as “frost drought” (Michaelis, 1934; Tranquillini, 1976; Sakai and Larcher, 1987; Körner, 2012). This water imbalance would promote death by needle desiccation in subalpine conifers. However, this phenomenon is not generally accepted as the leading cause for the existence of the alpine treeline (Mamet and Kershaw, 2013; Nakamoto et al., 2013). When accepted, there is no consensus on the environmental factors and the underlying physiological mechanisms involved, resulting in coexisting different interpretations about the majority presence of dry needles at the end of winter. Mayr et al. (2003, 2006) showed these drought signals as a consequence of frost drought that prevents water uptake and possibly amplified by repeated freeze-thaw cycles that increase the susceptibility of the xylem to embolism. Other studies have emphasized the role of leaf passive mechanisms for protection against desiccation as the thickness of the entire epidermal complex (hypodermis, epidermis, and cuticle) comparing populations with an extreme altitudinal difference (Baig and Tranquillini, 1976). These tools would comprise key factors to understand the recurrence of wilted and brown needles in the treeline specimens. Specifically, the main protective barrier of the leaves is the cuticle, a cutin matrix impregnated and coated with cuticular waxes whose primary function is to prevent uncontrolled water loss (Riederer and Schreiber, 2001; Schuster et al., 2017; Bueno et al., 2019).

The role of the cuticle is critical when stomata remain closed to reduce transpiration, such as during prolonged winter drought, as occurs with evergreen plant species in cold environments. Under these conditions, plant transpiration is determined by water diffusion through passive systems such as the cuticle (Gil-Pelegrín, 1993). Michaelis (1934), who first raised this idea, proposed that an inadequate “ripening” of the cuticle caused the greater transpiration rate during winter in the treeline. This phenomenon would be caused by the shortening of the vegetative period as a result of the temperature reductions at these altitudes. The increased cuticular water loss rate could explain the “frost drought” damage suffered by specimens at the treeline during the winter (Michaelis, 1934). Otherwise, Hadley and Smith (1983, 1986) proposed that the extreme abrasion of the cuticle (e.g., wind blow, ice crystals, or snow) caused the excessive water loss in the treeline more than an inadequate maturation during summer.

Therefore, few studies with different but not excluding explanations have investigated the role of passive needle mechanisms in protecting tree line conifers from winter water loss (Sakai and Larcher, 1987; Körner, 2012). Specifically, there is a lack of studies comparing very precise measurements of cuticular properties and cuticular waxes with minimum needle conductance (*g*_min_) in populations with small altitudinal differences. To shed light on this, the cuticular transpiration barrier was studied in the transition from subalpine *Pinus uncinata* forests (thereafter Forest) to alpine tundra at the upper limit of the species in the Pyrenees (thereafter Krummholz). This upper limit of *P. uncinata* was selected here as an example of the ecotones formed by conifers in the temperate mountains of the northern hemisphere.

In this study, we revisited Michaelis’ hypothesis (1934) and, thus, tested whether (i) specimens of *P. uncinata* living in the Krummholz display higher values of *g*_min_ than those specimens living in the Forest and (ii) whether changes in *g*_min_ between Krummholz and Forest are associated with changes in cuticle thickness and quantitative/qualitative changes in cuticular composition. To test these hypotheses, we have measured *g*_min_ at 25°C, cuticle thickness, and chemical composition for specimens of *P. uncinata* grown both at the Krummholz and Forest populations.

## Materials and Methods

### Study Site

**FIGURE 1:**
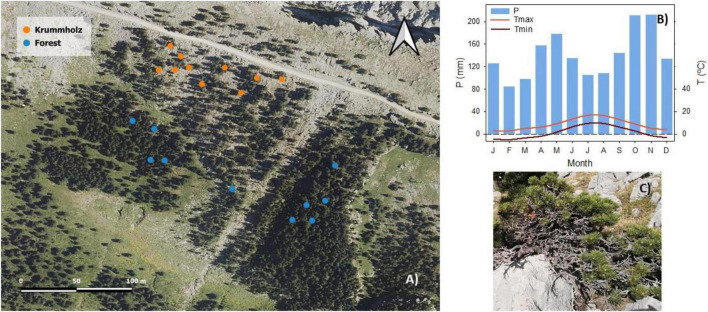
**(A)** Orthoimage obtained from the Spanish National Plan for Aerial Orthophotography (PNOA) corresponding to the year 2018 of Sierra de Las Cutas in the Spanish Pyrenees. Each point in the image corresponds to an individual (*n* = 10 per population). **(B)** Climograph of the study site, obtained from the climatic data of the Goriz Refuge located in the Ordesa y Monte Perdido National Park (Huesca, Spain) collected during the years 1981–2018. The monthly average precipitation (P), the monthly average maximum (Tmax), and minimum (Tmin) temperature are shown (Serrano-Notivoli et al., 2018). **(C)** Specimen of *Pinus uncinata* with the characteristic Krummholz mat shape.

Twigs of *P. uncinata* were collected on October 30th of 2019 in Sierra de las Cutas (Ordesa y Monte Perdido National Park, Huesca, Spain). The study was carried out in two nearby populations of a *P. uncinata* in the Spanish Pyrenees, the so-called Forest (42°38′10′′N, 0°03′07′′W, 2,150 m a.s.l.) and Krummholz (42°38′15′′ N, 0°03′12′′ W, 2,200 m a.s.l.) Figure 1A.

Both populations are found on calcareous soil with an average annual precipitation of 1,698 mm and an average winter snow cover of 63.2 cm. Although in the study area the snowfall is assured, there are periods with little snow cover due to the strong wind and continental conditions (see Supplementary Figure 1A). The coldest month is February with a minimum average temperature (T_min_) of −4.8°C and a maximum average temperature (T_max_) of 3°C; the warmest month is July with a T_min_ of 9.7°C and a T_max_ of 16.8°C (Figure 1B). Due to the altitude and soil porosity, the trees of the forest reached 7.6 ± 1.3 m in height and those of Krummholz 0.44 ± 0.01 m at their highest points. Tree height was measured with a Bitterlich relascope.

The analysis of the main climatic parameters that define the study site has been carried out based on the information collected by the Goriz observatory during the years 1981–2018. The refuge, with a slope, aspect and elevation very similar to those of the sampling area, is located at an altitude of 2,215 m a.s.l. on the southern slopes of the Monte Perdido massif located in the Ordesa y Monte Perdido National Park (Serrano-Notivoli et al., 2018).

The specimens selected as models of the typical monopodial bearing of the species were located within the central band of the pine forest that develops on the southern slope of the Sierra de las Cutas. Specifically, south-exposed, current-year needles from the first whorl of branches were sampled in Forest population. The collection of shoots in the Krummholz forms (see Figure 1C and Supplementary Figure 1B) was carried out in the central part of the tree, avoiding its most peripheral parts, damaged by local factors (permanence of snow and shading, especially).

### Minimum Needle Conductance

Five fascicles (two needles per fascicle) per tree from 10 trees were collected in each one of the studied populations and were fully hydrated in a humid chamber overnight before the measurements. The water-saturated weight (SW) was determined for each group of five fascicles using an analytical balance (MC-1 AC210S, Sartorius; precision 0.1 mg), and the dry weight (DW) was obtained after oven drying the samples at 90°C for 24 h. The fresh weights (FW) during needle-drying experiments were used to calculate the relative water deficit (RWD) according to the following:


$$\mathrm{RWD}=1-\frac{\mathrm{FW}-\mathrm{DW}}{\mathrm{SW}-\mathrm{DW}}$$

A flatbed scanner (CanoScan LiDE 120, Canon, Tokyo, Japan) was used to obtain high-resolution images of the needles and using the ImageJ image analysis software (Wayne Rasband/NIH, Bethesda, MD, United States) needle area was measured. Total needle surface area (*A*_needle_) was estimated from measurements of needle width (*w*) and length (*l*), and it was assumed that this area approximates a semi-cylinder:


$$A_{\mathrm{needle}}=\pi \,{\left(\frac{w}{2}\right)}^{2}+\left(\frac{w}{2}\right)\,\;\pi \,l+w\,l$$

Minimum needle conductance (*g*_min_) was determined from the consecutive weight loss of desiccating needles in darkness and at close to 0% relative humidity, following the methodology described by Bueno et al. (2020). The same needles used for RWD were used for *g*_min_ measurement.

g_min_ is the lowest conductance a needle can reach when stomata are maximally closed due to desiccation. Water-saturated needles were sealed with high melting paraffin wax (Paraplast Plus, Leica Biosystems, IL, United States). Subsequently, the sealed needles were placed in a closed container with a temperature of approx. 25°C. The air temperature and humidity were monitored using a digital thermo-hygrometer (Testoterm 6010, Testo, Lenzkirch, Germany). Silica gel (Envirogel, London, United Kingdom) was used to control the moisture, obtaining a relative humidity close to 0%. The weight of desiccating needles was determined as a function of desiccation time using an analytical balance (MC-1 AC210S, Sartorius, Gotinga, Germany; precision 0.1 mg). The transpiration rate (*J*) was calculated from the change in fresh weight (ΔFW) with time (*t*) divided by the *A*_*needle*_:


$$J=\frac{\Delta \,\mathrm{FW}}{\Delta \,t\times A_{\mathrm{needle}}}$$

The cuticular water conductance (*g*) was calculated from the transpiration rate (*J*) divided by the driving force for water loss from the outer epidermal cell wall to the surrounding atmosphere. The driving force for the vapor-based conductance corresponds to the difference between the saturation concentrations of water vapor at the temperature of the needle (*C*_wv sat needle_) and the surrounding atmosphere (*C*_wv sat air_) multiplied by the water activity in the epidermal apoplast (α_apo_) and the atmosphere (α_air_):


$$g=\frac{J}{\left(\alpha_{\mathrm{apo}}\times C_{\mathrm{wv}\,\mathrm{sat}\,\mathrm{needle}}\right)-\left(\alpha_{\mathrm{air}}\times C_{\mathrm{wv}\,\mathrm{sat}\,\mathrm{air}}\right)}$$

The water activity of the atmosphere (α_air_) over silica gel is nearly zero. The water activity in the apoplast adjacent to the inner side of the cuticle (α_apo_) is assumed to be close to one. Thus, the active driving force for cuticular transpiration in the setup used was the saturation concentration of water vapor at actual needle temperature (*C*_wv sat needle_). The corresponding water vapor saturation concentrations at needle temperature were derived from tabulated values (Nobel, 2009). The cuticular water conductance at a given dehydration point was plotted vs. the respective RWD.

### Anatomical Measurements

**FIGURE 2:**
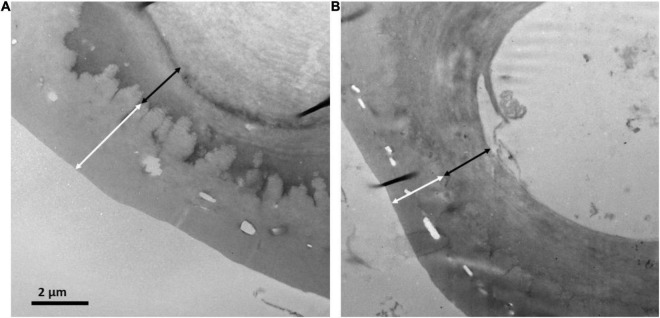
Transmission electron micrographs of the cuticle and outer cell wall for needles of *P. uncinata* grown at **(A)** Forest and **(B)** Krummholz populations. The white arrows represent cuticle thickness and the black arrows represent cell wall thickness.

Sections of 1 mm × 1 mm of 6 needles from each population were cut and processed for anatomical measurements as described in Peguero-Pina et al. (2016). Semi-thin (0.8 μm) and ultrathin (90 nm) cross-sections were cut with an ultramicrotome (Reichert and Jung model Ultracut E). Semi-thin cross-sections were stained with 1% toluidine blue and viewed under a light microscope (Optika B-600TiFL, Optika Microscopes, Ponteranica, Italy). Ultrathin cross-sections were contrasted with uranyl acetate and lead citrate and viewed under a transmission electron microscope (H600, Hitachi, Tokyo, Japan). Light and electron microscopy images were analyzed with ImageJ software^1^ to determine needle anatomical characteristics (Figure 2). Light micrographs were used to measure the thickness of the epidermis and hypodermis. Electron micrographs were used to measure the thickness of the cuticle and the outer cell wall of epidermal cells. Each anatomical trait was measured on six different fields of view per section, that is a total of 36 fields of view per population.

### Chemical Analyses of Cuticular Waxes

This section is taken *verbatim* from Bueno et al. (2020). Cuticular waxes were extracted by dipping the whole needles twice into trichloromethane (≥99.8%, Roth) at room temperature for 1.5 min. *N*-tetracosane (C_24_; ≥99.5%, Sigma-Aldrich) was added as an internal standard, and the solutions were reduced to dryness under a gentle flow of nitrogen. Dry cuticular wax samples were derivatized with *N,O*-bis(trimethylsilyl)trifluoroacetamide (BSTFA, Marchery-Nagel) in pyridine (≥99.5%, Roth) at 70°C for 30 min. Quantification of cuticular wax compounds was performed with a gas chromatograph equipped with a flame ionization detector and an on-column injector (7890A, Agilent Technologies). Separation of compounds was carried out on a fused silica capillary column (DB1-ms; 30 m length × 0.32 mm inner diameter, 0.1 μm film thickness, Agilent Technologies) with hydrogen as a carrier gas. The temperature program consisted of injection at 50°C for 2 min, raised by 40°C min^–1^ to 200°C, held at 200°C for 2 min, and then raised by 3°C min^–1^ to 320°C and held at 320°C for 30 min. Qualitative analysis was carried out using a gas chromatograph equipped with a mass spectrometric detector (5975 iMSD, Agilent Technologies) following the same gas chromatographic conditions but using helium as the carrier gas. Cuticular wax compounds were identified by comparing a query mass spectrum with reference mass spectra in a library *via* spectrum matching and quantitated against the internal standard.

### Statistical Analyses

The normality of data distribution was tested with Shapiro–Wilk test. To check equality of variances we applied Fisher’s F-test and the Mann – Whitney U test for those non-normally distributed. Student’s *t*-tests were used to compare values of *g*_min_, anatomical traits, and cuticular wax composition between Forest and Krummholz populations. *g*_min_ and anatomical traits means are presented along with their standard error and wax composition mean values are presented along with their standard deviation. All statistical analyses were performed in the R software environment (version 4.0.0, R Core Team, 2020).

## Results

**FIGURE 3:**
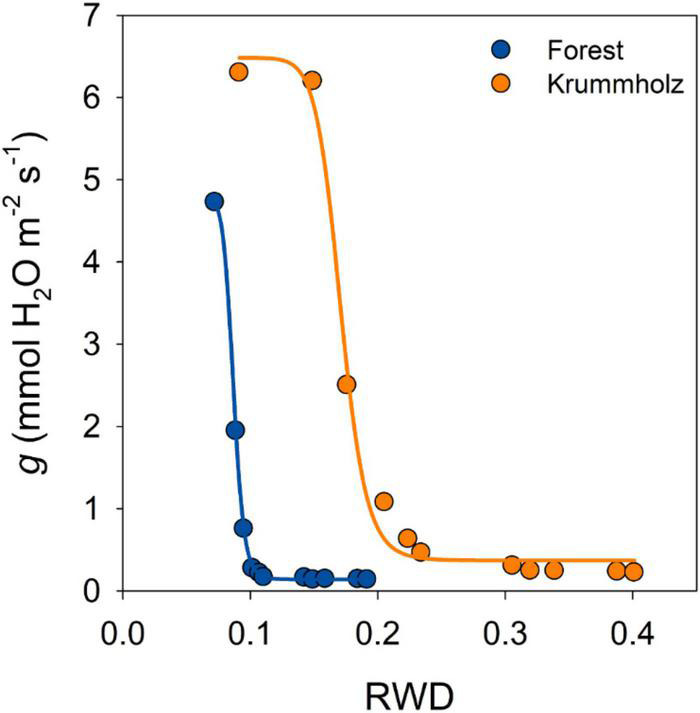
Cuticular water conductance (*g*) as a function of the relative water deficit (RWD) for a single representative tree of each one of the studied populations of *P. uncinata* (Forest and Krummholz). A sigmoidal four-parameter curve is fitted to guide the eye. The transition between the declining stage and the plateau stage of needle conductance represents stomatal closure. After maximum stomatal closure, needle conductance remains constant, representing the minimum needle conductance (*g*_min_).

**FIGURE 4:**
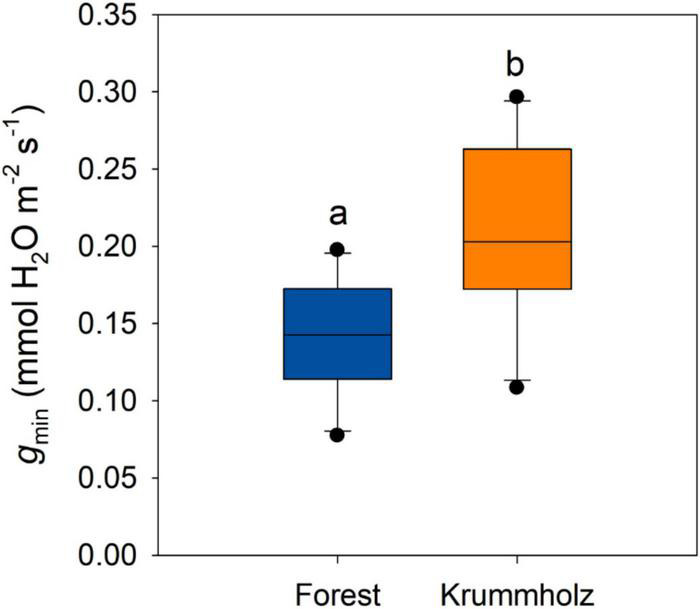
Minimum leaf conductance (*g*_min_) for needles of *P. uncinata* grown at Forest and Krummholz populations, obtained from drying curves at 25°C. Different letters indicate statistically significant differences (*p* < 0.05) between both populations.

The first stage of drying curves for needles of both populations was characterized by high conductance that decreased with leaf dehydration until reaching a plateau of constant needle conductance values (*g*_min_), after reaching maximum stomatal closure (Figure 3). The value of *g*_min_ for needles from Krummholz (0.210 ± 0.018 mmol H_2_O m^–2^ s^–1^) was 1.5-fold higher (*p* = 0.005, Figure 4) than that measured for Forest needles (0.142 ± 0.012 mmol H_2_O m^–2^ s^–1^).

**FIGURE 5:**
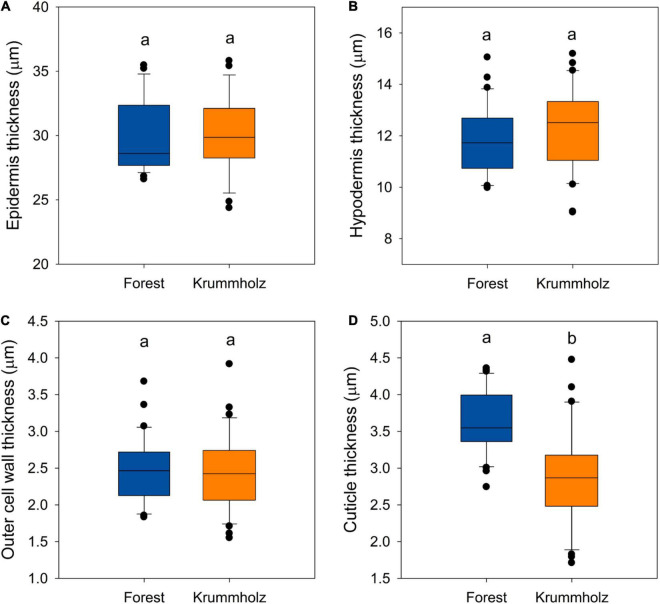
The thickness of the **(A)** epidermis, **(B)** the hypodermis, **(C)** the outer cell wall of the epidermis, and **(D)** the cuticle for needles of *P. uncinata* grown at Forest and Krummholz populations. Different letters indicate statistically significant differences (*p* < 0.05) between both populations.

Regarding anatomical traits, it should be highlighted that cuticle was thicker (*p* < 0.001, Figures 2, 5) in needles from the Forest population (3.62 ± 0.66 μm) in comparison to the Krummholz population (2.87 ± 0.43 μm). By contrast, the thicknesses of the epidermis, hypodermis, and the outer cell wall of epidermal cells did not differ between both populations (*p* = 0.879, *p* = 0.352, and *p* = 0.802, respectively, Figure 5). Needle length was 5.2 ± 0.2 cm in Forest and 3.1 ± 0.1 cm in Krummholz (data not shown).

**FIGURE 6:**
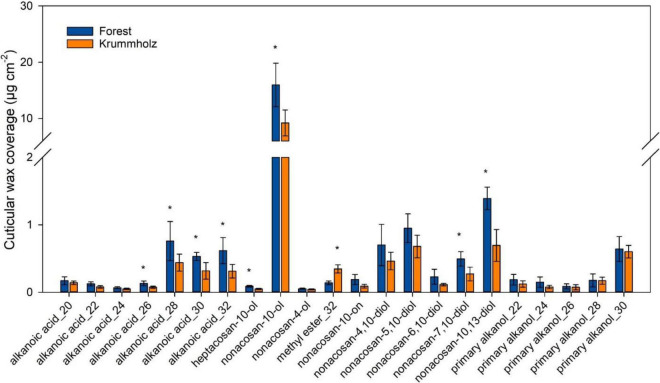
Contribution of single compounds to the cuticular wax coverage of *P. uncinata* needles grown at Forest and Krummholz populations (*n* = 5; mean ± standard deviation). Asterisks indicate statistically significant differences (*p* < 0.05) between both populations.

The cuticular wax load was higher for needles from Forest (35.2 ± 6.3 μg cm^–2^) compared with those from Krummholz (22.9 ± 5.1 μg cm^–2^). Non-acosan-10-ol was the major cuticular wax constituent for both Forest (15.9 ± 3.8 μg cm^–2^) and Krummholz (9.2 ± 2.3 μg cm^–2^), values statistically different when compared between populations (*p* = 0.017) (Figure 6). Among the other identified compounds, none is exclusive to one population, however, the relative contributions of single compounds in each of the populations do not always follow the same pattern. Thus, long-chain alkanoic acids between C_28_ and C_32_, heptacosan-10-ol, non-acosan-7,10-ol and non-acosan-10,13-ol abundancy was higher in Forest needles; short-chain alkanoic acids from C_20_ to C_24_ and primary alkanols do not shown differences between populations; and C_32_ methyl ester was more abundant at the Krummholz population (Figure 6).

## Discussion

Our results demonstrate that the specimens of *P. uncinata* living in the transition with alpine tundra and the upper limit of the species (Krummholz population) displayed higher values of *g*_min_, lower values of cuticle thickness and total cuticular wax load than those living in the adjacent subalpine forest. Nonetheless, we still stress that these findings should be interpreted carefully and should not be extrapolated to other plant species and environments since there is strong evidence that *g*_min_ in some species correlates neither with cuticular wax load nor cuticular thickness (Schreiber and Riederer, 1996; Riederer and Schreiber, 2001; Schuster et al., 2017; Bueno et al., 2020). In addition, it must be taken into account that the values of *g*_min_ have a great variation and can differ among plant organs or even between the two sides of a single leaf (Diarte et al., 2020; Márquez et al., 2022).

Among the components found in cuticular waxes, non-cacosan-10-ol is the most abundant, being about ten times more abundant than the others (Figure 6). Non-acosan-10-ol is the main constituent of the tubular microcrystalline wax aggregates that have been described for the stomatal antechambers of coniferous needles, conferring an additional resistance to stomatal transpiration (Jetter and Riederer, 1994; Dommisse et al., 2007). Thus, this compound would be discarded in the comparison of cuticular waxes strictly related to *g*_min_. However, it results in the fact that wax production is generally lower in Krummholz needles.

Michaelis (1934) proposed that a greater residual transpiration rate during winter is produced due to an inadequate maturation of the cuticle in the needles from the treeline. This hypothesis was revisited by Wardle (1974) and Tranquillini (1979), again suggesting this idea as a general cause to explain the existence of an upper treeline. In fact, Baig and Tranquillini (1976) showed that residual transpiration in needles from upper limit specimens of *Pinus cembra* and *Picea abies* was higher than those measured in specimens living in the lower levels of the subalpine forest. In addition, these authors also revealed that the upper limit specimens displayed lower thickness values of the entire epidermal complex (hypodermis, epidermis, and cuticle), which for the cuticle was attributed to a shorter summer developmental time.

We have obtained similar results to those in Baig and Tranquillini (1976) in terms of differences in *g*_min_ and cuticle thickness, which should reinforce the seminal hypothesis by Michaelis (1934). Further, the contribution of the different wax fractions to the cuticular transpiration barrier of *P. uncinata* is still to be determined. In fact, most of the studies concerning Krummholz physiology at the upper treeline do not confirm Michaelis’ hypothesis (Hadley and Smith, 1983, 1986; Grace, 1990). Some of them associated the damage found in Krummholz needles to direct winter damage rather than inadequate development during the vegetative period. In this sense, Grace (1990) reported higher winter transpiration in needles of *Pinus sylvestris* in United Kingdom at treeline but he did not find signs of inadequate cuticular development in needles. Besides that, Hadley and Smith (1983, 1986) also found a higher *g*_min_ in the subalpine conifers constituting the upper treeline in Wyoming (United States), but they explained such difference through the abrasion in the cuticle caused by harsh environmental conditions that can occur in the treeline during winter. Nevertheless, our measurements were carried out during autumn in current-year needles, which minimizes the possibility of winter degradation factors, such as abrasion and stomatal dysfunction, contributing to the higher *g*_min_ and thinner cuticle thickness in Krummholz trees (Hadley and Smith, 1983, 1986). Instead, our results favor the Michaelis (1934) hypothesis of inadequate cuticle development.

However, it cannot be assumed that worldwide alpine treelines result from inadequate cuticle development (Körner and Paulsen, 2004). Thus, *Pinus pumila* shows no correlation between cuticle characteristics and needle mortality at treeline when comparing two Krummholz populations growing at high altitude with different wind exposures (Nakamoto et al., 2012). Otherwise, Anfodillo et al. (2002) studying *Picea abies* and *Pinus cembra* in the Dolomites along a broad altitudinal gradient, found that these species have thicker cuticles at higher elevations. However, in our study, it is hard to assume that a climatic gradient associated with changes in altitude can modify in such way the cuticle of both growth forms. This would be a big difference compared to the study of Baig and Tranquillini (1976) in which the differences in altitude between populations are approximately 1,000 m, while here it is close to 50 m. The effect of several and continuous alterations of the apical dominance by continuous over pruning, modify the growth pattern from the typical tall, erect timber-sized tree to the stunted Krummholz. Furthermore, it could also be suggested that this over pruning alters the carbon allocation patterns and/or carbohydrate status of the needle so that trees have less carbon available to develop their cuticles, suggesting a trade-off between carbon assimilation and water loss with *g*_min_ (Machado et al., 2021). This may be a factor that should be explored to explain this modified functioning.

The differences found in the present study in terms of *g*_min_ and cuticle thickness between Forest and Krummholz populations may increase the risk of “frost drought” in the treeline (Michaelis, 1934; Tranquillini, 1976). However, other factors besides *g*_min_ and cuticle thickness should be considered when analyzing the possible existence of “frost drought” in the treeline. For instance, the occurrence of a high number of freeze-thaw events could promote massive embolism and needle desiccation during winter in the alpine treeline (Mayr et al., 2006). One might think that the continuous loss of shoots due to “frost drought” condition their stunted form significantly, modifying its nature and the performance of its needles. In this sense, one might wonder if the lower cuticle development, the smaller needle size, the lower cuticle thickness and the greater *g*_min_ in Krummholz specimens have a causal relationship, however, this is only a mere hypothesis that should be tested in further studies.

Therefore, the upper treeline is a complex phenomenon that probably cannot be explained from a single point of view, and more research is needed to provide a general explanation for its existence. Our study showed clear evidence that supports the inadequate development of needle cuticles as one of the factors that lead to increased transpirational water losses during winter and, consequently, a higher risk of suffering frost drought. However, other factors associated with the harsh conditions suffered by shoots during winter (i.e., cuticle abrasion or a high number of freeze-thaw events) may also increase the risk of “frost drought” and should be considered when analyzing the upper treeline.

## Data Availability Statement

The original contributions presented in the study are included in the article/Supplementary Material, further inquiries can be directed to the corresponding author.

## Author Contributions

EG-P, AB, and DA-F conceived the idea and collected the plant material. EG-P and JP-P conceptualized and supervised the project. AB, DA-F, JF, and DS-K contributed to the conception and design of the study. DA-F performed the minimum needle conductance measurements. JP-P and DA-F achieved the anatomical measurements and analyzed the data and wrote the first draft of the manuscript. AS performed the chemical analyses of cuticular waxes. All authors contributed to manuscript revision, read, and approved the submitted version.

## Conflict of Interest

The authors declare that the research was conducted in the absence of any commercial or financial relationships that could be construed as a potential conflict of interest.

## Publisher’s Note

All claims expressed in this article are solely those of the authors and do not necessarily represent those of their affiliated organizations, or those of the publisher, the editors and the reviewers. Any product that may be evaluated in this article, or claim that may be made by its manufacturer, is not guaranteed or endorsed by the publisher.

## Footnotes

1
http://rsb.info.nih.gov/nih-image/

## Supplementary Material

Supplementary Figure 1 Specimens of *Pinus uncinata* from Krummholz population in Sierra de Las Cutas, Spanish Pyrenees. **(A)** Krummholz tree at the upper limit of the species, partially covered with snow. **(B)** Krummholz specimen with signs of browning and lesions on the needles.
