# Supplementary figures and images for: Minimum Leaf Conductance (gmin) Is Higher in the Treeline of Pinus uncinata Ram. in the Pyrenees: Michaelis’ Hypothesis Revisited
Source: Front Plant Sci. 2022 Jan 24;12:786933. doi: 10.3389/fpls.2021.786933 (PMC8818696; doi:10.3389/fpls.2021.786933)

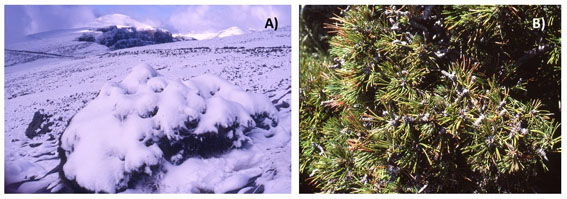

Supplement: Supplementary Figure 1 — Specimens of Pinus uncinata from Krummholz population in Sierra de Las Cutas, Spanish Pyrenees. (A) Krummholz tree at the upper limit of the species, partially covered with snow. (B) Krummholz specimen with signs of browning and lesions on the needles. [file Image_1.JPEG]
